# Inadequate Iodine Intake in Population Groups Defined by Age, Life Stage and Vegetarian Dietary Practice in a Norwegian Convenience Sample

**DOI:** 10.3390/nu10020230

**Published:** 2018-02-17

**Authors:** Anne Lise Brantsæter, Helle Katrine Knutsen, Nina Cathrine Johansen, Kristine Aastad Nyheim, Iris Erlund, Helle Margrete Meltzer, Sigrun Henjum

**Affiliations:** 1 Division of Infection Control and Environmental Health, Norwegian Institute of Public Health, Oslo 0403, Norway; helle.knutsen@fhi.no (H.K.K.); hellemargrete.meltzer@fhi.no (H.M.M.); 2 Department of Nursing and Health Promotion, Faculty of Health Sciences, OsloMet—Oslo Metropolitan University, Oslo 0310, Norway; nina_c_johansen@hotmail.com (N.C.J.); kristinen91@hotmail.com (K.A.N.); sigrun.henjum@hioa.no (S.H.); 3 Department of Chronic Disease Prevention, National Institute for Health and Welfare, Helsinki 00271, Finland; iris.erlund@thl.fi

**Keywords:** iodine status, urinary iodine concentration, probability of adequate iodine intake, iodine-supplement use, children, adolescents, elderly, pregnant, vegetarians, vegans

## Abstract

Inadequate iodine intake has been identified in populations considered iodine replete for decades. The objective of the current study is to evaluate urinary iodine concentration (UIC) and the probability of adequate iodine intake in subgroups of the Norwegian population defined by age, life stage and vegetarian dietary practice. In a cross-sectional survey, we assessed the probability of adequate iodine intake by two 24-h food diaries and UIC from two fasting morning spot urine samples in 276 participants. The participants included children (*n* = 47), adolescents (*n* = 46), adults (*n* = 71), the elderly (*n* = 23), pregnant women (*n* = 45), ovo-lacto vegetarians (*n* = 25), and vegans (*n* = 19). In all participants combined, the median (95% CI) UIC was 101 (90, 110) µg/L, median (25th, 75th percentile) calculated iodine intake was 112 (77, 175) µg/day and median (25th, 75th percentile) estimated usual iodine intake was 101 (75, 150) µg/day. According to WHOs criteria for evaluation of median UIC, iodine intake was inadequate in the elderly, pregnant women, vegans and non-pregnant women of childbearing age. Children had the highest (82%) and vegans the lowest (14%) probability of adequate iodine intake according to reported food and supplement intakes. This study confirms the need for monitoring iodine intake and status in nationally representative study samples in Norway.

## 1. Introduction

Iodine is an essential nutrient required for the synthesis of the thyroid hormones triiodothyronine and thyroxine, which are critical to fetal brain development and metabolic processes in the body throughout life [1]. Iodine deficiency, manifested as goiter, was prevalent in Norway, but disappeared around 1950 due to the introduction of iodine fortification of cattle fodder and ample milk consumption [2,3]. Despite eradication of goiter for many decades, inadequate iodine status has recently been reported in Scandinavia, Britain and many other European countries [4,5,6,7,8]. Iodine requirements increase during pregnancy and lactation, and inadequate iodine intake in pregnant and lactating women is of particular concern due to associations with impaired child development [9,10,11]. Both iodine deficiency and excess have adverse health consequences [12,13], highlighting the need for systematic monitoring of iodine status in all population groups [14]. Denmark initiated monitoring of iodine status more than 20 years ago, and the findings resulted in implementation of mandatory fortification of industrial (commercial) bread with iodized salt [15]. Other countries in Europe and worldwide have handled this issue in different ways or have ignored it [6,16,17,18,19]. 

Milk and seafood are the main dietary iodine sources in Norway, while the contribution from low levels in drinking water and iodine fortified table salt (<1%) is considered to be negligible [7,20]. Surveys have shown reduced consumption of milk and seafood during the last decades and reduced iodine content in milk, possibly due to more substances in the feed inhibiting iodine uptake [21,22,23]. 

Iodine from food is readily absorbed and ~92% is excreted in the urine, making urinary iodine concentration (UIC) a good indicator of recent iodine intake at the population level [24]. The World Health Organization (WHO) recommends using the median UIC from spot urine samples of children and mothers of childbearing age to describe the iodine status of a population [1]. Due to day-to-day variation in recent iodine intake and hydration status, the use of single spot urine samples to quantify individuals as having deficient or excess iodine intakes is likely to overestimate the true prevalence of deficient or excess intakes [25,26,27]. Calculating iodine intake from food and dietary supplements may provide a more complete understanding of usual iodine intake than iodine concentrations measured in spot urine samples alone [28,29], particularly in populations with few dietary sources. When the dietary intake assessment relies on short term intake, the impact of reporting errors and day-to-day variation can be reduced by adjustment of individual intake distribution for within- and between person variation [26,30].

Existing information on iodine status in subgroups of the Norwegian population is limited, particularly in young children, adolescents and the elderly, as well as in vegetarians and vegans; the latter intentionally eliminate the main iodine-containing foods such as milk and fish from their diet. The aim of this study was therefore to evaluate iodine status by assessing urinary iodine concentration and the probability of adequate iodine intake in subgroups of the Norwegian population defined by age, life stage and vegetarian dietary practice.

## 2. Materials and Methods

### 2.1. Population and Study Design

The study design is cross-sectional and participants were primarily recruited to an international study of dietary exposure to deoxinivalenol, a mycotoxin originating from cereals [31]. The informed consent for Norwegian participants included a statement allowing use of dietary information and urine samples to study other substances from food such as iodine. We recruited participants by convenience sampling among employees at two Norwegian research institutes, the Norwegian Institute of Public Health and the Norwegian Veterinary Institute. The recruitment period lasted from April 2014 to December 2014. We posted a call for participants, including information about the study and project workers’ contact information on the institutions’ internal websites. In order to recruit children, adolescents, the elderly, pregnant women and vegetarians, the information encouraged the employees to involve their children and extended family. We developed a simplified information leaflet for children. A total of 257 participants participated in the study of which data for 230 were included in the deoxinivalenol study [31]. In addition to those recruited for the deoxinivalenol study, we recruited 19 additional participants among students and employees at Oslo Metropolitan University from June to September 2015. The second recruitment followed the same principles as the first recruitment.

After giving informed, written consent, participants provided information about age, weight, height, dietary restriction, vegetarian dietary practice, smoking and other lifestyle habits in a short interview. Together with a project worker, they choose two days to record all food, drink and dietary supplements consumed in an open diary and to provide first morning void spot urine samples on the two mornings after completing the food records. Most participants (90%) collected data on two consecutive days.

### 2.2. Ethics

The study was conducted according to the guidelines laid down in the Declaration of Helsinki and written informed consent was obtained from all participants, including parental consent for children younger than 16 years of age. The Regional Committee for Medical and Health Research Ethics approved the study (REK 2014/207). 

### 2.3. Iodine Intake from Food and Supplements

Participants received an open food diary and information on how to record all food and drink items for two whole days (24-h food record). They received instruction to report all dietary supplements by name and manufacturer, and to record all food and drink items and all mixed dishes to the nearest gram (or mL). When a kitchen scale was not available, we asked participants to report consumed amounts in household measures and to include a description of the portion size (small, medium, large). For mixed dishes, we asked for the name of the dish and a list of single ingredients in addition to the total weight or household measure. 

Two trained research assistants coded all food and drink items in the food records manually. Food items were assigned a food number used in the Norwegian Food Composition Table [32]. If an item recorded by any participant was not present in the table, we assigned the number of a similar food item or from the international literature. The total number of food items in the Norwegian Food Composition Table with analytical iodine concentrations has been expanded in recent years. For items with missing iodine values, we assigned values based on ingredients and recipes. Finally, we used FoodCalc to calculate food, energy and nutrient intakes [33]. All food supplements were reported by brand name, producer and ingested dose. Using the information provided by producers and labels, we calculated the amount of iodine in µg/day contributed by iodine-containing supplements. This amount was added to the amount of iodine contributed by food (µg/day) to obtain total iodine intake. Total energy intake was evaluated to identify potential under- and over-reporting of food intake. We considered all the 276 food diaries to be valid.

### 2.4. Determination of Urinary Iodine and Creatinine

Fasting spot urine samples were collected on each of the two mornings following food recording. Participants received two 0.5 L plastic bottles with a wide opening and a double lid for collecting the samples. The urine samples were kept cold until collection by a project worker and distributed into vials stored at −20 °C until analysis. 

Urinary iodine concentrations were determined at the National Institute for Health and Welfare (THL) in Helsinki (Finland). Iodine measurements were carried out by inductively coupled plasma—mass spectrometry using an Agilent 7800 ICP-MS system (Agilent Technologies Inc., Santa Clara, CA, USA). In brief, 100 µL of urine sample was extracted by using ammonium hydroxide solution. Tellurium was used as an internal standard. On the ICP-MS, m/z = 127 was scanned for iodine determination. The limit of quantification (LOQ) was 2 µg/L and the linearity was excellent up to 1500 µg/L (r = 0.9999). Coefficient variation (CV%) was 2–3%. The National Institute of Standards and Technology (NIST) reference standard materials SRM2670a (with certified mass concentration value) and SRM3668 Levels 1 and 2 were used to ensure the accuracy of urinary iodine concentrations. The laboratory at THL participates in Ensuring the Quality of Urinary Iodine Procedures Program (EQUIP) organized by the Centers for Disease Control and Prevention (CDC) three times per year.

Urinary creatinine concentrations were analyzed at the Department of Drug Analysis, Norwegian Institute of Public Health, according to an accredited method using a colorimetric assay (modified kinetic Jaffe method) on a Beckman Coulter AU680 analyzer (Beckman Coulter Inc., Brea, CA, USA). In brief, the method is a reaction with alkaline picrate forming a red-orange complex. The color intensity is directly proportional to the creatinine concentration and measured spectrophotometrically at 505 nm. The LOQ is 0.2 mml/L. We present urinary iodine concentrations as µg iodine per L urine and as µg iodine per g creatinine. 

### 2.5. Definitions

According to WHO, a population is defined as iodine sufficient when the median UIC is between 100 μg/L and 299 µg/L in school-age children and non-pregnant individuals. In addition, no more than 20% of the UIC samples should be lower than 50 µg/L [1]. Assuming 90% absorption and 1.5 L urine volume, an UIC of 100 μg/L corresponds to a daily intake of 150 µg iodine. The iodine requirement increases in pregnancy and a median UIC between 150 and 249 µg/L defines sufficient iodine intake in this group [1]. Excessive iodine intake is defined by WHO as median UIC at or above 300 µg/L in children and non-pregnant adults, and at or above 500 µg/L in pregnant women [1].

The recommended daily intake (RDI) is the intake level of a nutrient considered to meet the requirement of nearly all healthy individuals over time, while the estimated average requirement (EAR) is the intake level estimated to meet the requirement of half of the healthy individuals in a group. In Norway, the RDI for iodine is based on the Nordic Nutrition Recommendations 2012 (NNR12) [34] and is 90 µg/day in children aged 2–5 years, 120 µg/day in children aged 6–9 years, 150 µg/day in adolescents aged 10–17 years and adults (≥18 years), 175 µg/day during pregnancy, and 200 µg/day during lactation [34]. The RDI for pregnant and lactating women is lower in NNR12 than the corresponding RDI issued by WHO, which is 250 µg/day for pregnant and lactating women [1]. The dietary reference value for iodine derived by the European Food Safety Authority is 200 µg/day for both pregnant and lactating women [35]. The US Institute of Medicine has established EARs for all life stages and gender groups: 65 µg/day for children 1–8 years, 73 µg/day for children 9–13 years, 95 µg/day for adolescents 14–18 years, 160 µg/day for pregnant women, and 95 µg/day for adult and elderly men and women [36].

### 2.6. Statistics

Data processing and analyses were performed using IBM SPSS statistics version 23 (IBM Corp., Armonk, NY, USA) and STATA 14 (StataCorp, College Station, TX, USA). We assessed variable distribution using visual inspection of plots (histograms and box plots). *p*-values < 0.05 are considered statistically significant. We present normally distributed data as mean and standard deviation and non-normally distributed data as median, 95% confidence intervals (95% CI), interquartile range (IQR), and 25 and 75 percentiles (P25, P75). We used the Wilcoxon matched pair signed-rank test to examine differences, and Spearman rank correlation and intraclass correlation coefficients (ICC) to assess agreement between paired measurements. ICC was obtained by linear mixed model regression and is the ratio of between-person variation and total variation (between-person + within person variation). 

We calculated iodine intakes from food and supplements recorded in the two 24-h food records (µg/day) and used the two 24-h iodine intakes to estimate usual iodine intake and the probability of adequate iodine intake by the Institute of Medicine’s probability approach [37,38]. The probability approach takes into account the random within- and between-person variance and estimates a more reliable long-term intake than just the two days. The probability approach makes use of the estimated usual intake data and EAR for each individual to construct a probability curve, and each individual’s intake is plotted along this function. When the probability of adequacy for a nutrient is averaged across individuals, it is equivalent to the population prevalence of adequacy expressed as a percentage [37,38].

UIC was not normally distributed and values were log-transformed when used in mean (least square) regression. However, we used median regression analysis (quantile regression) with non-transformed UIC as the dependent variable to evaluate predictors of median UIC. Median regression is less sensitive to outliers than mean regression and estimates changes to the median of the dependent variable (rather than the mean), conditional on the values of the independent variable [39]. We included seven categorical variables as independent variables: sex (female vs. male), age (three categories vs 18–64 years as reference), smoking (yes vs. no) and vegan (yes vs. no) and present the adjusted coefficients and 95% confidence intervals for differences in median UIC for independent variable categories relative to the reference categories. Only variables with *p*-values < 0.10 were included in the final model.

## 3. Results

### 3.1. Participant Characteristics

The study sample included 156 (57%) female and 120 male participants. Vegetarian dietary practice has multiple definitions [40]. The current study included 44 vegetarians comprising two subgroups. Participants who reported no intake of meat or fish, but included eggs and/or milk, were denoted OL-vegetarians (*n* = 25), while participants who reported no meat or animal-derived food items were denoted vegans (*n* = 19). Table 1 shows participant characteristics by non-overlapping groups according to age, life-stage and vegetarian practice. The OL-vegetarian group included one child, two adolescents, 21 adults and one elderly, while the vegan group comprised adults only. Pregnant women and vegetarians more often reported use of iodine-containing supplements than the other groups. Of the supplement users (*n* = 26), 23 used multivitamin-mineral supplements and three used kelp-supplements. Iodine supplements contributed on average 147 µg/day in iodine-supplement users. Five participants reported daily smoking and five reported occasional smoking. 

### 3.2. Urinary Iodine Concentration (UIC) and Iodine Intake from Food and Supplements

The measured UICs as well as the calculated iodine intakes on day 1 and day 2 did not differ between the two days (Supplementary Table S1). We therefore used the mean of the two days for evaluation of urinary iodine and calculated iodine intake. The Spearman correlation coefficient for UIC between the two morning urines was 0.57 for iodine expressed as µg/L and 0.65 for iodine expressed as µg/g creatinine. The correlation between the day 1 and 2 was 0.45 for iodine from food and 0.75 for iodine from supplements. ICC for urinary iodine was 0.61 for UIC expressed as µg/L and 0.57 for UIC expressed as µg/g creatinine, while ICC for iodine from food was 0.36.

The median and 95% CI for UIC was 101 (90, 100) µg/L in all participants combined. Children had the highest and vegans, the elderly and pregnant women had the lowest median UIC (Table 2). Children had the smallest and OL-vegetarians the largest 95% CIs to median UIC. IQRs for UIC were 67 µg/L in children, 56 µg/L in adolescents, 65 µg/L in adults, 61 µg/L in the elderly, 64 µg/L in pregnant women, 88 µg/L in OL-vegetarians and 37 µg/L in vegans. Adjusting for urinary creatinine increased the median UIC for pregnant women and the elderly, while vegans still had the lowest median. According to the WHO criteria for evaluating iodine intake from UIC at the group level [1], iodine intake is sufficient when median UIC is above 100 µg/L in non-pregnant and above 150 µg/L in pregnant individuals. The median UIC was close to the cut-off for adults and below the cut-off for the elderly, pregnant women and vegans. Although it is not correct to assume that all individuals with UIC lower than cut-off are iodine deficient, the highest proportions were seen in the elderly (70%), pregnant women (89%) and vegans (95%) (Table 2).

Excessive iodine intake (defined as UIC at or above 300 µg/L) was evident for 12 individuals, of which two were OL-vegetarians and one was a vegan. These three had the highest observed UICs and all reported using kelp-supplements. According to the supplement labels, one serving would provide 150 µg iodine, while the observed UICs in the two morning spots were 1409 and 1093 µg/L for person a, 1253 and 75 µg/L for person b, and 846 and 598 µg/L for person c. The remaining nine individuals with UIC above 300 µg/day included three children (UIC range in individual day spot samples from 297 to 491 µg/L), three adolescents (UIC range 301 to 646 µg/L) and three adults (UIC range from 227 to 776 µg/L). Of these, one adolescent and one adult reported iodine supplement use. In the pregnant women, no participants had excessive iodine intake, i.e., UIC above 500 µg/L. 

In all participants combined, the median (5th, 95th percentile) calculated iodine intake from food was 103 (29, 273) µg/day and the corresponding intake from food and supplements combined was 112 (31, 286) µg/day (Table 3). The majority of participants in all groups had iodine intake below the recommended iodine intake [34]. 

### 3.3. Estimated Usual Iodine Intake and Probability of Adequate Iodine Intake

In all participants combined, the median (P25, P75) estimated usual iodine intake was 101 (75, 150) µg/day and the mean iodine intake was 119 µg/day. The mean probability of adequate iodine intake was 60% in all participants combined (Table 4). Children had the highest (82%) and vegans the lowest probability (14%) of adequate iodine intake, while the probability of adequacy was 47% in pregnant women and ~60% in adolescents, the elderly and ovo-lacto vegetarians (Table 4). No participants had calculated or usual iodine intake above the safe upper level, i.e., 200 µg/day for children 1–3 years, 300 µg/day for children 4–8 years, 600 µg/day for children 9–13 years, 900 µg/day for adolescents 14–18 years, 1100 µg/day in adults 19 years and older [36].

### 3.4. Predictors of Urinary Iodine Concentrations

The results showed moderate to high correlation between calculated iodine intake and UIC. In all participants combined, the correlation with total iodine intake was 0.46 for UIC expressed as µg/L and 0.47 for UIC expressed as µg/g creatinine. The corresponding correlation coefficients in subgroups ranged from 0.35 in adolescents to 0.46 in children, 0.50 in pregnant women, 0.55 in adults, 0.56 in vegans, 0.71 in the elderly, and 0.79 in ovo-lacto vegetarians (*p* < 0.05 for all). Correlations were lower for estimated usual iodine intake than for calculated iodine intake. 

In all participants combined, UIC was higher in iodine-containing supplement users than non-users (*p* < 0.001). Although iodine-supplement use was more prevalent in women, women had lower UIC than men (*p* < 0.001). In non-pregnant women of childbearing age (18–47), median (95% CI) UIC was 71 (61, 94) µg/L, while the UIC in adult men was 110 (72, 120) µg/L. UIC did not differ between non-pregnant women of childbearing age and pregnant women (*p* = 0.33).

**Figure 1:**
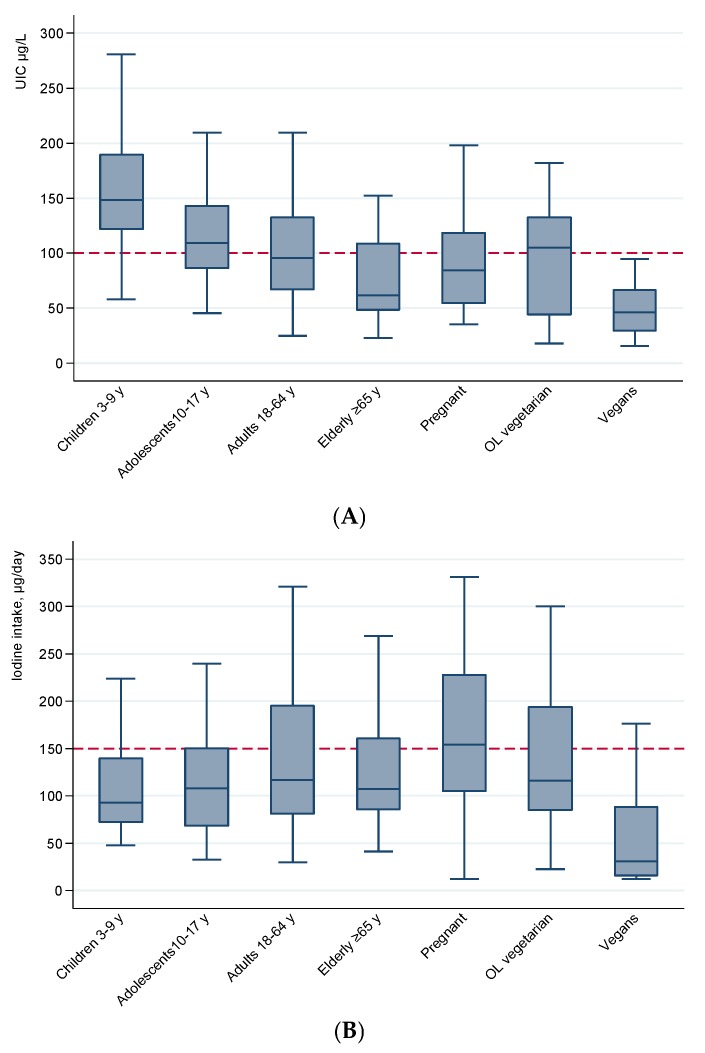
Urinary iodine concentration (UIC) µg/L (**A**) and calculated iodine intake (**B**) by non-overlapping subgroups defined by age, life stage and dietary practice. Box plot details: the horizontal lines indicate the median; the box indicates the interquartile range (IQR) (25th percentile to 75th percentile); the whiskers represent observations within 1.5 times the IQR. Outliers with values more than 1.5 times the IQR away from the box are not shown. The dashed line in A represents the WHO cut-off for sufficient iodine intake in non-pregnant groups. The corresponding cut-off in pregnant women is 150 µg/L. The dashed line in B represents recommended iodine intake in children >9 years, non-pregnant adults and the elderly. Recommended intakes in the other groups are 90 µg/day for children 2–5 years, 120 µg/day for children 6–9 years, and 175 µg/day for pregnant women [34].

Pairwise comparison of UIC between the non-overlapping population groups showed that median UIC was higher in children (*p* < 0.01) and lower in the elderly (*p* = 0.05) and vegans (*p* < 0.01) than in adults (Figure 1A). However, UIC did not differ between adolescents and adults (*p* = 0.19) or between OL-vegetarians and non-vegetarian adults (*p* = 0.50).

Pairwise comparison of total iodine intake between the groups showed that iodine intake was higher in children than in adults (*p* = 0.029), higher in pregnant women than in non-pregnant, non-vegetarian women (*p* < 0.001), and lower in vegans than in non-vegetarian adults (*p* < 0.001). There was no difference in iodine intake between adolescents, the elderly or OL-vegetarians and non-vegetarian adults (all *p* > 0.05) (Figure 1B). 

**Figure 2:**
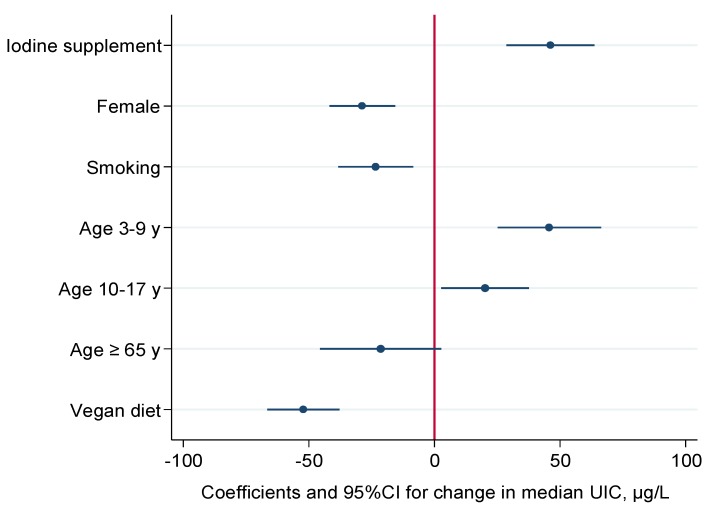
Plot showing the change in median urinary iodine concentration (UIC) µg/L, coefficients and 95% confidence interval for supplement use, sex, smoking, age and vegan dietary practice. *N* = 276.

In multiple regression analyses of UIC (log-transformed), calculated iodine intake from food and iodine supplement use as independent variables, with additional adjustment for age, sex and energy intake, explained 40% of the variance in UIC (β = 0.43 for a 10 µg increase in iodine intake and β = 0.51 for iodine supplement use). In multiple quantile regression evaluating change (µg/L) in median UIC we did not include iodine from food, but examined differences in median UIC in supplement vs. non-users, three age groups (children, adolescents and the elderly) vs. adults (reference), female vs. male (reference), smokers vs. non-smokers (reference) and vegans vs. non-vegans (reference). Compared to adults, children (3–9 years) and adolescents (10–17 years) had significantly higher median UIC and the elderly (≥ 65 years) had lower median UIC. Female participants, smokers and vegans had significantly lower UIC than male participants, non-smokers and non-vegans (Figure 2). Including iodine from food in the model did not substantially change the differences in UIC between these groups (data not shown).

Regressing creatinine-adjusted UIC as the dependent variable (urinary iodine as µg/g creatinine rather than µg/L) resulted in comparable coefficients for the differences between iodine-supplement vs. non-supplement users, children vs. adults, and vegans vs. non-vegans. However, the differences in UIC between non-smokers vs. smokers, adults vs. adolescents, and adults vs. elderly disappeared when UIC were adjusted for creatinine (data not shown). 

### 3.5. Sources of Iodine

**Figure 3:**
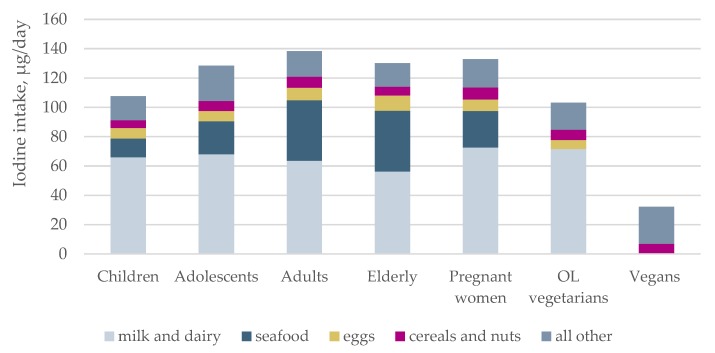
The mean contribution to iodine intake (µg/day) from different food groups in children, adolescents, adults, the elderly, pregnant women, ovo-lacto (OL) vegetarians and vegans.

**Figure 4:**
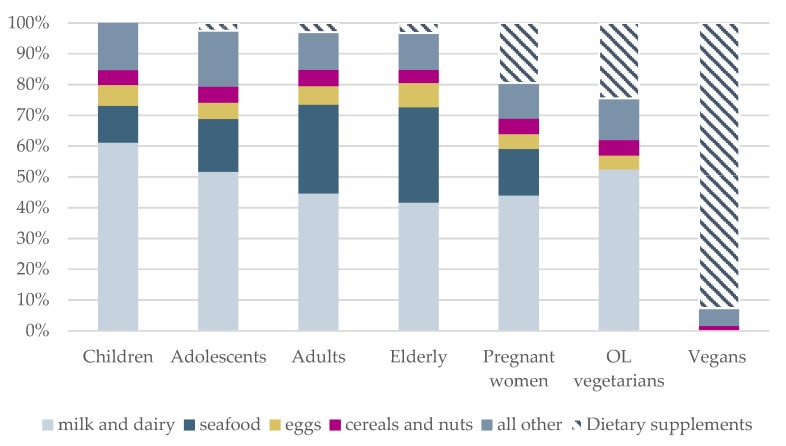
The contribution (%) to total iodine from food groups and dietary supplements in children, adolescents, adults, the elderly, pregnant women, ovo-lacto (OL) vegetarians and vegans.

Evaluation of food intakes and iodine sources showed that milk, including yoghurt and milk products, was the main source of iodine in all subgroups except vegans (Figure 3), contributing on average 40–60% of iodine from food. In vegans, the main source of iodine was iodine-containing supplements (Figure 4).

## 4. Discussion

The present study evaluated iodine status in groups defined by age, life stage and vegetarian dietary practice. The results should be interpreted with caution as the study population was not nationally representative and the groups were small. According to the WHO criteria for median UIC [1], children (3–9 years), adolescents (10–17 years), adults (18–64 years), and OL-vegetarians had adequate iodine intake, while the elderly (≥65 years), pregnant women, non-pregnant women of childbearing age, and vegans had inadequate iodine intake. These findings were confirmed by the estimated usual iodine intake and the probability of adequacy. The proportion of participants with UIC below WHO cut-off values for population median was particularly high in pregnant women (89%) and vegans (95%). The calculated total iodine intake was lower than the recommended daily intake [34] for the majority of individuals in all groups. The estimated usual iodine intake and the low probability of iodine adequacy intake confirmed insufficient intake. The probability of adequacy was particularly low in pregnant women (47%) and vegans (14%). According to calculated total iodine intake, excessive iodine intake was rare, while UIC revealed some individuals with excessive intake, with particularly high concentrations in individuals who reported use of kelp supplements. High UIC in non-iodine supplement users, e.g., children, may be explained by high intake of milk. 

Our results add to the evidence that inadequate iodine intake is not confined to low-income countries [41]. Inadequate iodine status has been documented in pregnant and lactating Norwegian women earlier [7,8,42], while studies in children, adolescents, the elderly and vegetarians have been lacking. Because maternal iodine status is of particular importance for fetal brain development, the high proportion of pregnant women with low urinary iodine and low probability of adequate iodine intake is of special concern. The low UIC in non-pregnant women of childbearing age is also of high concern. Moderate iodine deficiency has been associated with adverse cognitive outcomes in children [9,11]. Observational studies within the Norwegian Mother and Child Cohort study indicate adverse neurocognitive outcomes in children born to mothers with iodine intakes below 160 µg/day [10,43]. 

We found that children had the highest median UIC, the highest usual iodine intake and the lowest probability of inadequate iodine intake. Adequate iodine nutrition has been reported in school-age children in Sweden [44], Portugal [45], and Australia [46] and a number of countries globally, simultaneously to finding inadequate iodine status in pregnant women in the same populations [16,47,48,49]. As milk is a main iodine source in countries without mandatory salt iodization, higher consumption of milk and dairy products in younger age groups explains why children are iodine sufficient while their mothers are not. The importance of milk for iodine intake is well recognized, and individuals that for various reasons do not include milk in their diet will be at high risk of insufficient iodine intake. 

To the best of our knowledge, this is the first study to evaluate iodine intake in Norwegian vegetarians. The number of people adopting a vegetarian diet is increasing worldwide [50]. We categorized vegetarians into OL-vegetarians, who included eggs and milk in their diet, and vegans, who excluded all animal derived food items. While iodine status did not differ between OL-vegetarians and non-vegetarians of comparable age, iodine intake was particularly inadequate in vegans, with the exception of those taking iodine-containing supplements. With milk and white fish being the only available substantial iodine sources in the diet, it is obvious that individuals omitting these food items need to obtain iodine from supplements or from salt fortified with more iodine than what is currently available in Norway. It is important to be aware that popular milk alternatives (based on non-dairy ingredients like soy or oats) do not contain iodine [51]. The low probability of adequate iodine intake in vegans is in line with results from other studies in vegans and vegetarians in Europe [52,53,54,55] and the US [56]. Although most of the studies, like our study, are based on relatively small study samples (*n* = 15–70), the results of all the studies, independent of sample size, corroborate insufficient iodine intake or status in vegans. Vegans clearly rely on iodine supplements to secure recommended iodine intake. However, the form of supplements must be considered and our finding of UIC values above 1000 µg/L in kelp-supplement users highlight that standardization of the iodine content in kelp supplements is problematic. Because there is large variation in the iodine content in different types of kelp, kelp-supplements should be avoided. 

Comparison of population UIC in spot samples with established UIC cut-offs is the recommended method for evaluation of iodine adequacy at the population level, while no existing biomarker accurately reflects iodine intake at the individual level [27]. As UIC reflects short term intake and is liable to hydration status, calculation of the probability of adequate iodine intake may be a more accurate measure of iodine insufficiency and excess [25,26]. A study in sex- and life-stage specific subgroups in the US population found lower prevalence of iodine inadequacy using the probability of adequate iodine intake method than using the UIC cut-off method [30]. The authors concluded that consideration of dietary iodine intake from all sources might provide a more complete understanding of population prevalence of iodine inadequacy and excess, and thus better inform dietary guidance than consideration of UIC alone.

There is diurnal variation in iodine excretion and iodine measured in morning urine samples has lower intra-individual variation than iodine measured in spot urine samples collected at random time points [57]. It must be taken into account that although restricting urine samples to morning urine reduce intra-individual variation, UIC has been shown to be lower in the morning than in afternoon and evening samples [58]. However, there is little difference between UIC in fasting morning urine and 24-h urine samples and morning urine samples are acceptable for assessing iodine status at the group level [59]. The inter- and intra-individual variation in UIC is caused by differences in iodine intake as well as by large variation in fluid intake. Creatinine is excreted at a relatively constant rate and creatinine adjustment (e.g., the μg iodine/g creatinine ratio) has been suggested to minimize the variation in UIC in µg/L caused by variation in the urinary volume [60]. In this study, regression of median UIC expressed as µg/g creatinine rather than as µg/L attenuated the differences between age and sex, indicating that the differences observed for UIC expressed as µg/L mainly reflected differences in urine production. As creatinine excretion varies with age, sex and protein intake, the usefulness of the iodine/creatinine ration has been questioned [29,59,61].

Iodization of salt has successfully reduced iodine deficiency worldwide in the last decades and WHO encourages universal mandatory salt iodization [62]. Public health authorities in some countries consider salt a problematic vehicle for iodine fortification as high salt intake is linked to hypertension and cardiovascular disease [63]. With ongoing campaigns to reduce salt intake, iodine fortification of salt may counteract this effort. Health authorities in Denmark and Australia have implemented mandatory use of iodized salt in bread as a means of supplementing the whole population, rather than just relying on iodized household salt, and this has resulted in improved iodine status in most groups of these populations [15,64]. Another measure to improve iodine status encouraged by WHO is the use of iodine-containing supplements [65]. Use of iodine-containing supplements results in higher UIC [66], and this was also seen in the current study. For pregnant women, it should be noted that use of iodine-containing supplements should be initiated prior to pregnancy, as initiation in pregnancy may be too late to counteract the adverse associations observed for low maternal iodine intake and child neurodevelopment [10,43].

The strengths of this study include two spot urine samples and two detailed 24-h food diaries for all participants. A major limitation of the current study is the relatively small sample size, particularly in the subgroups comprising the elderly, OL- vegetarians and vegans. Another limitation is that participants are not representative of the Norwegian population as they were primarily recruited among employees at two research institutions. The participants therefore are likely to have better socio-economic status (SES) and include fewer smokers than the general population. Given that individuals with high SES are likely to be more health conscious and have better diets than individuals with lower SES, it is not likely that iodine status will be better in the general population than in this study population. However, the results need to be replicated in larger, better designed and nationally representative samples. 

## 5. Conclusions

This cross-sectional study in population groups defined by age, life stage and vegetarian dietary practice, showed that children had median UIC above the WHO cut-off and also had the highest probability of adequate iodine intake according to dietary assessment. However, the elderly, pregnant women, non-pregnant women of childbearing age and vegans had median UIC below the WHO cut-off and had the lowest probability of adequate iodine intake. The current study shows that dietary sources of iodine do not secure adequate iodine intake in all groups of the population, and use of iodine containing supplements is required to meet iodine recommendations in vegans and individuals who cannot meet iodine recommendations from diet alone. However, consideration needs to be given to the form of supplement as kelp supplements can lead to iodine excess. Although the results must be interpreted with caution, this study confirms the need for the monitoring of iodine intake and status in nationally representative samples of the Norwegian population.

## Figures and Tables

**Table 1:** Characteristics of the study population by non-overlapping subgroups, total *n* = 276.

Subgroups	*n*	Sex (Male) *n* (%)	Age, Years Mean ± SD	Weight, kg Mean ± SD	Supplementary Iodine *n* (%)
Age and life-stage groups					
Children 3–9 years	47	30 (64)	6.3 ± 1.9	23 ± 5.6	0
Adolescents 10–17 years	46	17 (37)	13.4 ± 2.3	50 ± 12.7	1 (2.2)
Adults 18–64 years	71	34 (48)	42.1 ± 11.4	73 ± 12.7	2 (2.8)
Elderly 65+ years	23	11 (48)	70.7 ± 6.7	72 ± 11.3	2 (8.7)
Pregnant women	45		33.0 ± 4.6	66 ± 8.2	11 (24)
Vegetarians combined	44	14 (32)	34.2 ± 13.1	66 ± 15.1	10 (23)
Vegetarian groups					
Ovo-Lacto vegetarians ^1^	25	11 (44)	37.2 ± 15.7	64 ± 15.9	5 (20)
Vegans ^2^	19	3 (16)	30.1 ± 7.1	67 ± 14.4	5 (16)

^1^ Ovo-Lacto vegetarians are individuals who reported no meat and fish in their diet, but they reported intake of eggs and/or milk products; ^2^ Vegans are individuals who reported no animal products in their diet.

**Table 2:** Urinary iodine concentration (UIC) expressed as µg/L and as µg/g creatinine, in all participants combined and by non-overlapping subgroups.

Subgroup	*n*	UIC ^1^ µg/L	UIC^1^ µg/g Creatinine	Inadequate Iodine Intake ^2^	Excessive Iodine Intake ^3^
		Median (95% CI)	Median (95% CI)	*n* (%)	*n* (%)
All participants	276	101 (90, 110)	92 (85, 100)	148 (54)	12 (4.3)
Children 3–9 years	47	148 (129, 145)	143 (130, 174)	5 (11)	3 (6.4)
Adolescents 10–17 years	46	109 (90, 123)	76 (64, 92)	19 (41)	3 (6.5)
Adults 18–64 years	71	96 (81, 113)	77 (66, 89)	38 (54)	3 (4.2)
Elderly 65+ years	23	62 (51, 97)	91 (74, 127)	16 (70)	0
Pregnant women	45	84 (62, 107)	101 (78, 135)	40 (89)	0
Ovo-Lacto Vegetarians	25	105 (50, 129)	84 (63, 101)	12 (48)	2 (8.0)
Vegans	19	46 (32, 62)	47 (25, 48)	18 (95)	1 (5.3)

^1^ All data are the mean of urinary iodine concentration for day 1 and day 2; ^2^ WHO cut-off for adequate iodine intake: 100 µg/L for school-aged children and non-pregnant adults, 150 µg/L for pregnant women [1]; ^3^ WHO cut-off for excessive iodine intake: 300 µg/L in school aged children and non-pregnant adults, 500 µg/L in pregnant women.

**Table 3:** Calculated daily iodine intake (µg/day) from two-day food records in all participants combined and by non-overlapping subgroups.

Subgroup	*n*	Iodine from Food Only ^1^	Total Iodine ^2^	Total Iodine below RDI ^3^
		Median (P25, P75)	Median (P25, P75)	*n* (%)
All participants	276	103 (69, 150)	112 (77, 175)	182 (66)
Children 3–9 years	47	93 (72, 139)	93 (72, 139)	27 (57)
Adolescents 10–17 years	46	106 (68, 150)	108 (68, 151)	33 (72)
Adults 18–64 years	71	116 (80, 195)	117 (81, 195)	47 (66)
Elderly 65+ years	23	107 (85, 161)	107 (85, 161)	16 (70)
Pregnant women	45	118 (90, 176)	154 (105, 228)	26 (58)
Ovo-Lacto Vegetarians	25	86 (50, 134)	116 (85, 194)	16 (64)
Vegans	19	26 (15, 42)	31 (15, 88)	17 (90)

^1^ Mean of calculated intakes on day 1 and day 2; ^2^ Total iodine intake is iodine from food aggregated with iodine contributed by dietary supplements; ^3^ RDI: Recommended daily intake according Nordic Nutrition Recommendation 2012 [34]: 90 µg/day for children 2–5 years, 120 µg/day for children 6–9 years, 150 µg/day for children >9 years, adults and the elderly, and 175 µg/day for pregnant women.

**Table 4:** Estimated usual iodine intake ^1^ and probability of adequate iodine intake in all participants combined and by non-overlapping subgroups.

Subgroup	*n*	Estimated Usual Iodine Intake, µg/d		Probability of Adequacy (%) ^2^
		Median (P25, P75)	Mean (SD)	Mean (SD)
All participants	276	101 (75, 150)	119 (66)	60 (41)
Children 3–9 years	47	89 (77, 120)	100 (34)	82 (28)
Adolescents 10–17 years	46	92 (67, 133)	115 (74)	61 (43)
Adults 18–64 years	71	109 (76, 149)	122 (63)	63 (39)
Elderly 65+ years	23	112 (76, 168)	121 (58)	61 (37)
Pregnant women	45	154 (107, 225)	161 (73)	47 (42)
Ovo-Lacto Vegetarians	25	102 (64, 175)	119 (65)	60 (41)
Vegans	19	57 (15, 90)	64 (59)	14 (32)

^1^ Estimated usual intake according to the best linear unbiased predictor calculated from the two individual 24-h food records for each individual [36,37]; ^2^ Mean probability of adequate iodine intake according to the estimated average requirement according to age- and lifestage [36].

## Supplementary Materials

The following are available online at http://www.mdpi.com/2072-6643/10/2/230/s1, Table S1: Urinary iodine concentration (UIC) in two morning spot urine samples (µg/L) and total iodine intake from food and supplements by two 24-h food records (µg/day) in all participants and by non-overlapping subgroups.
