# Supplementary material for: Inadequate Iodine Intake in Population Groups Defined by Age, Life Stage and Vegetarian Dietary Practice in a Norwegian Convenience Sample
Source: Nutrients. 2018 Feb 17;10(2):230. doi: 10.3390/nu10020230 (PMC5852806; doi:10.3390/nu10020230)
Supplement: Supplementary file 1 [file nutrients-10-00230-s001.pdf]

Supplementary Material

Supplementary Table S1. Urinary iodine concentration (UIC) in two morning spot urine samples (µg/L) and total iodine intake from food and supplements by two 24-hour food records (µg/day) in all participants and by non-overlapping subgroups.

Subgroup	n	UIC day 1	UIC day 2	*p*-value [2]	Iodine intake day 1	Iodine intake day 2	*p*-value [2]
		Median (P25, P75)	*Median (P25, P75)*		*Median (P25, P75)*	*Median (P25, P75)*	
All participants	276	93 (55, 140)	93 (55, 156)	0.46	97 (65, 188)	107 (61, 175)	0.45
Subgroup							
Children 3–9 years	47	148 (114, 188)	149 (96, 190)	0.94	83 (64, 143)	104 (63, 140)	0.36
Adolescents 10–17 y	46	102 (73, 139)	112 (78, 167)	0.05	91 (57, 177)	105 (57,183)	0.40
Adults 18–64 years	71	86 (51, 138)	92 (56, 155)	0.70	111 (72, 194)	105 (63, 181)	0.65
Elderly 65+ years	23	60 (35, 174)	67 (51, 83)	0.52	116 (73, 184)	115 (54, 158)	0.39
Pregnant women	45	75 (49, 97)	87 (49, 133)	0.23	156 (87, 239)	140 (102, 229)	0.20
OL-vegetarians[3]	25	93 (37, 120)	75 (46, 132)	0.84	103 (62, 207)	92 (74, 139)	0.31
Vegans[3]	19	50 (37,70)	28 (22, 52)	0.07	29 (15, 90)	22 (16, 87)	0.45

[1] Calculated total iodine intake from food and supplements. [2] *P*-value by the Wilcoxon signed rank test of two related samples. [3] Ovo-Lacto vegetarians (OL-vegetarians) are individuals who reported no meat and fish, but reported intake of eggs and/or milk products in their diet. Vegans are individuals who reported no animal products in their diet.
